# The prevalence of avascular necrosis in patients treated with chemotherapy for testicular tumours

**DOI:** 10.1054/bjoc.2001.2155

**Published:** 2001-11

**Authors:** A M Cook, A S K Dzik-Jurasz, A R Padhani, A Norman, R A Huddart

**Affiliations:** Academic Department of Radiotherapy and Oncology, Department of X-Ray, Department of Computing and Information, The Royal Marsden NHS Trust, and Institute of Cancer Research, Downs Road, Sutton, Surrey,M2 5PT, UK

**Keywords:** testicular cancer, avascular necrosis, chemotherapy steroids

## Abstract

To study the prevalence of avascular necrosis in patients receiving chemotherapy for testicular cancer we invited 103 consecutive patients treated by chemotherapy to attend for MRI scan of the hips. Four of 47 (9% (CI 2–20%)) patients scanned and 4/103 (3.8% (CI 1–10%)) of patients invited to participate in the study had evidence of avascular necrosis. As not all patients in the study had completed the at risk period this equates to a 3-year actuarial risk of 6.3% (95% confidence limits (CI) 2.4–16.1). These data suggest that avascular necrosis is an uncommon but significant complication of chemotherapy including steroids as anti-emetics. © 2001 Cancer Research Campaign http://www.bjcancer.com


*British Journal of Cancer* (2001) **85**(11), 1624–1626
© 2001 Cancer Research Campaign
doi: 10.1054/ bjoc.2001.2155, available online at http://www.idealibrary.com on IDEAL
http://www.bjcancer.com

### The prevalence of avascular necrosis in patients treated with chemotherapy for testicular tumours

**AM Cook[1], ASK Dzik-Jurasz[2], AR Padhani[2], A Norman[3] and RA Huddart[1]**

[1]Academic Department of Radiotherapy and Oncology; [2]Department of X-Ray; and [3]Department of Computing and Information, The Royal Marsden NHS Trust, and Institute of Cancer Research, Downs Road, Sutton, Surrey SM2 5PT, UK

**Summary** To study the prevalence of avascular necrosis in patients receiving chemotherapy for testicular cancer we invited 103 consecutive patients treated by chemotherapy to attend for MRI scan of the hips. Four of 47 (9% (CI 2–20%)) patients scanned and 4/103 (3.8% (CI 1–10%)) of patients invited to participate in the study had evidence of avascular necrosis. As not all patients in the study had completed the at risk period this equates to a 3-year actuarial risk of 6.3% (95% confidence limits (CI) 2.4–16.1). These data suggest that avascular necrosis is an uncommon but significant complication of chemotherapy including steroids as anti-emetics. © 2001 Cancer Research Campaign http://www.bjcancer.com

**Keywords**: testicular cancer; avascular necrosis; chemotherapy steroids

Avascular necrosis (AVN) has been described as a complication of combination chemotherapy, especially where it includes intermittent high dose corticosteroids (Gogas and Fennelly, 1996; Harper et al, 1984; Hui and Wiernik, 1996; Mould and Adam, 1983; Perloff and Lesnick, 1980; Socie′ et al, 1994; Thorne et al, 1981; Tombolini et al, 1992). We have recently reported five patients treated with chemotherapy for testicular tumours who developed symptomatic avascular necrosis following chemotherapy with steroids used as an antiemetic (Cook et al, 1999).

In this study we aim to assess the prevalence of avascular necrosis in patients who have received first line chemotherapy for testicular tumours.

### MATERIALS AND METHODS

All patients who had completed at least two cycles of first line chemotherapy in the 5 years prior to the start of the study who have remained relapse free and had at least 6 months follow up (March 1993–September 1997) were asked to participate in this study. After giving written consent they completed a questionnaire regarding hip pain and risk factors for AVN and were asked to have magnetic resonance imaging (MRI) scan. T1 (TE = 12 ms, TR = 600 ms, 150 degree flip angle, 5 mm slice thickness to no intergap, 192 × 256 matrix size, 3 excitations) and short time in version recovery (TE = 60 ms, TR = 4230 ms, TI = 150 ms, 5 mm slice thickness, 198 × 256 matrix size, 1 excitation matrix size) sequences were performed in the coronal plane through the hip joints, the field of view included the proximal femora and pelvis.

Time to AVN was calculated using the methods of Kaplan and Meier (1958) using the time from chemotherapy to diagnosis of AVN as the endpoint. Patients with no AVN were censored at the date of enrolment to the study.

### RESULTS

Of the 103 eligible patients who were contacted, 63 returned questionnaires. The median follow-up of patients invited for screening was 32 months (range 7.1–65 months) their characteristics are described in Table 1. In this cohort were two of the cases we had previously reported to have bilateral AVN of the hips (Cook et al, 1999). To avoid bias they were included in the analysis as they satisfied the eligibility criteria for the study. One further patient informed us that he had recently been diagnosed as having AVN of both hips on MRI at another hospital. These images were reviewed and the diagnosis confirmed. Sixteen patients did not have MRI scans because of failure to attend or being unsuitable due to metallic implants.

Of the 44 patients imaged one was found to have avascular necrosis in both hips with changes indicative of active disease. This patient had been experiencing bilateral hip pain for several weeks, which had previously been undiagnosed. All four patients with AVN had had bilateral hip pain. Two additional patients described bilateral hip pain but did not attend for MRI scanning. Seven patients

**Table 1** Patient characteristics

| | |
|---|---|
| Number of patients sent questionnaire | 103 |
| Age (median) | 30 (range 16–59) |
| Chemotherapy regime | |
| BEP 2 cycles | 21 |
| BEP 3–4 cycles | 52 |
| C-BOP BEP | 19 |
| BOP 2 cycles | 5 |
| *Steroid dose (mean (range)) | 2.25 g (0.76–3.86 g) |

*Converted to equivalent dose of prednisolone.
B, Bleomycin; C, Carboplatin; P, Cisplatin: E, Etoposide; O, vincristine.

*Received 12 March 2001*
*Revised 2 August 2001*
*Accepted 18 September 2001*

*Correspondence to:* RA Huddart

AM Cook, current address: Staffordshire Centre, North Staffordshire NHS Trust, Royal Infirmary, Princess Road, Hartshill, Stoke-on-Trent, Staffordshire ST4 7LN, UK

AR Padhani, current address: Mount Vernon Hospital, Rickmansworth Road, Northwood, Middlesex HA6 RN, UK

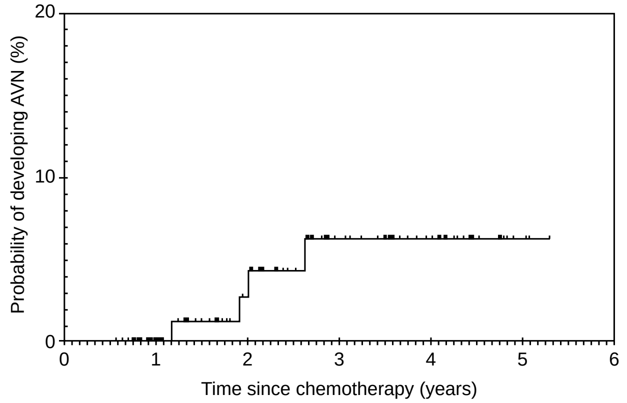

Number at risk

AVN 103 87 62 37 21 3

**Figure 1** Actuarial risk of developing avascular necrosis following chemotherapy for testicular cancer described unilateral or non specific hip pain; six attended for MRI, none of whom had avascular necrosis. The frequency of AVN in the patients undergoing MR examination was 4/47 (9%, CI 2–20). AVN was diagnosed, at a median of 21 months (range 14–32 months) after commencement of chemotherapy.

If it is assumed that all affected patients were symptomatic and that all symptomatic patients attended for MR examination, the overall prevalence was 4/103 (3.8%, CI 1–10%). As not all patients had completed the at risk period this equates to a 3-year actuarial risk of 6.3% (95% confidence limits (CI) 2.4–16.1) (Figure 1).

The affected patients received BEP chemotherapy either four cycles or three cycles and received total steroid doses equivalent to 2.97 g, 2.49 g, 3.3 g and 1.45 g of prednisolone respectively (mean 2.55 g). Thus three of the four patients received above the mean dose (2.25 g) of steroids administered. These three patients had no known predisposing factors for AVN. The patient who received the equivalent of 1.45 g of prednisolone had a high alcohol intake, a known predisposing factor to AVN (Mirzai et al, 1998). However, the number of events are too small to draw any meaningful conclusions.

### DISCUSSION

Our results demonstrate that patients receiving chemotherapy for testicular tumours, when dexamethasone is used as an anti-emetic, may develop avascular necrosis. It can be presumed that the same would be true for other patients treated for solid tumours.

We have tried to estimate the importance of this complication by documenting the frequency with which it occurs in one specific group of patients with testicular tumours. The prevalence of AVN in this study may be as high as 9% (CI 2–20%). All affected patients in our study were symptomatic and, if we assume that patients with hip pain were more likely to return questionnaires and attend for MR examination then the prevalence could be quoted as 3.8% (CI 1–10%). On actuarial analysis this equates to a 3-year risk of 6.3% (95% CI 2.4–16.1). It does seem more likely that patients with symptoms would have attended for MR examinations and indeed 13 of the 47 patients (28%) who did attend had noticed some hip pain. The prevalence of pain in patients not returning questionnaires is not known. However, it is interesting to note that 3 of the 16 patients (19%) who returned questionnaires but did not have MRI had described hip pain and in 2 of these the pain was bilateral.

A potential criticism of this study is that of bias in the sample. It was the recent diagnosis of AVN in several patients (Cook et al, 1999) that persuaded us to look at the problem in more depth. We tried to eliminate bias by approaching all eligible patients who had received chemotherapy within a recently treated cohort. Two of the previously diagnosed patients were included as they were treated within the time period of the study. Before undertaking this study we had not been aware of the diagnosis of AVN in the other two patients. If our previously identified patients are excluded the incidence of AVN is still in the order of 2% (2/101).

There is a recognized association of avascular necrosis and steroid use. Avascular necrosis has been reported when steroids are used with chemotherapy as part of the treatment regime for tumours such as lymphomas (Gogas and Fennelly, 1996; Hui and Wiernik, 1996; Mould and Adam, 1983; Perloff and Lesnick, 1980; Socie′ et al, 1994; Thorne et al, 1981; Tombolini et al, 1992). Our data and that from scattered case reports suggest that chemotherapy with steroids used as anti-emetics carries a significant risk of AVN. With the exception of the one case where there was a history of alcohol abuse all patients developing AVN in this study and in our previous report (Cook et al, 1999) received above our the mean steroid dose (2.25 g), raising the question as too whether there could be a dose response relationship for the development of AVN. However as AVN has been described in patients treated with chemotherapy without steroids (Harper et al, 1984) it is possible the situation may be more complex than a pure steroid related effect.

Relatively high doses of dexamethasone in combination with 5HT3 antagonists have been shown to be a high effective regime for controlling nausea in highly emetogenic regimens and are being used routinely as such.

This data has implications for this practice.

- As there is little evidence for an anti-emetic dose response for the dexamethasone when it is used with 5HT3 antagonists; it would be worth considering whether equivalent antiemesis could be obtained with a lower risk of AVN by using protocols with lower steroid doses.
- Patients who developed hip pain after chemotherapy warrant further investigation with plain radiographs and/or MRI.
- Patients receiving high dose steroids as anti-emetics should be warned of the risk of avascular necrosis so as to be able to make an informed choice.

### ACKNOWLEDGEMENTS

This work was undertaken in The Royal Marsden NHS Trust who received a proportion of its funding from the NHS Executive; the views expressed in this publication are those of the authors and not necessarily those of the NHS Executive. This work was supported by the Institute of Cancer Research, the Bob Champion Cancer Trust and the Cancer Research Campaign.

### REFERENCES

Cook A, Patterson H, Nicholls J and Huddart R (1999) Avascular necrosis in patients treated with BEP chemotherapy for testicular tumours. *Clin Oncol* **11**: 126–127

© 2001 Cancer Research Campaign 

Gogas H and Fennelly D (1996) Avascular necrosis following extensive chemotherapy and dexamethasone treatment in a patient with advanced ovarian cancer: case report and review of the literature. *Gynecol Oncol* **63**: 379–391

Harper P, Trask C and Souhami R (1984) Avascular necrosis of bone caused by combination chemotherapy with corticosteroids. *BMJ* **288**: 267–268

Hui L and Wiernik P (1996) Avascular necrosis of bone after adult acute lymphocytic leukemia treatment with methotrexate, vincristine, L-asparaginase, and dexamethasone (MOAD). *Am J Hematol* **52**: 184–188

Kaplan EL and Meier P (1958) Non-parametric estimation from incomplete observations. *J Am Stat Assoc* **53**: 457–481

Mirzai R, Chang C, Greenspan A and Gershwin ME (1998) Avascular necrosis. *Compr Ther* **24**: 251–255

Mould J and Adam N (1983) The problem of avascular necrosis of bone in patients treated for Hodgkin's disease. *Clin Radiol* **34**: 231–236

Perloff M and Lesnick G (1980) Avascular Necrosis of the Femoral head: Association with Adjuvant Chemotherapy for Breast Carcinoma. **64**: 361–362

Socie′ G, Selimi F, Sedel L, Frija J, Devergie A, Esperou Bourdeau H, Ribaud P and Gluckman E (1994) Avascular necrosis of bone after allogeneic bone marrow transplantation: clinical findings, incidence and risk factors. *Br J Haematol* **86**: 624–628

Thorne J, Evans W, Alison R and Fournasier V (1981) Avascular necrosis of bone complicating treatment of malignant lymphoma. *Am J Med* **71**: 751–758

Tombolini V, Capua A and Pompili E (1992) Avascular necrosis of the femoral head after treatment of Hodgkin's disease. *Acta Oncologica* **31**: 64–65

*© 2001 Cancer Research Campaign*
